# CyNetworkBMA: a Cytoscape app for inferring gene regulatory networks

**DOI:** 10.1186/s13029-015-0043-5

**Published:** 2015-11-11

**Authors:** Maciej Fronczuk, Adrian E. Raftery, Ka Yee Yeung

**Affiliations:** Institute of Technology, University of Washington, Tacoma, 98402 WA USA; Department of Statistics, University of Washington, Seattle, 98195 WA USA

## Abstract

**Background:**

Inference of gene networks from expression data is an important problem in computational biology. Many algorithms have been proposed for solving the problem efficiently. However, many of the available implementations are programming libraries that require users to write code, which limits their accessibility.

**Results:**

We have developed a tool called CyNetworkBMA for inferring gene networks from expression data that integrates with Cytoscape. Our application offers a graphical user interface for networkBMA, an efficient implementation of Bayesian Model Averaging methods for network construction. The client-server architecture of CyNetworkBMA makes it possible to distribute or centralize computation depending on user needs.

**Conclusions:**

CyNetworkBMA is an easy-to-use tool that makes network inference accessible to non-programmers through seamless integration with Cytoscape. CyNetworkBMA is available on the Cytoscape App Store at http://apps.cytoscape.org/apps/cynetworkbma.

**Electronic supplementary material:**

The online version of this article (doi:10.1186/s13029-015-0043-5) contains supplementary material, which is available to authorized users.

## Background

Networks in the form of directed and undirected graphs are commonly used to model complex interactions between biological entities in a living organism. The construction of gene regulatory networks from omics data is a fundamental problem in computational biology [1]. Recent advances in high-throughput methods have enabled us to rapidly quantify expression levels of large numbers of genes at low cost. This new abundance of big data sources highlights unique challenges in turning such data into useful information on regulatory relationships. The high dimensionality of expression data has spurred the search for robust and computationally efficient network inference algorithms.

Network inference is a computationally intensive process and different approaches have been shown to work well with different types of data sets [2, 3]. Bayesian networks [4] have been used to construct gene networks using gene expression data [5, 6]. Algorithms based on Bayesian networks that integrate multiple data sources have also been developed. For example, Zhu et al. integrated gene expression, DNA variation, DNA protein binding, protein metabolite interaction, and protein protein interaction data using Bayesian networks [7, 8]. Other methods rank edges based on correlation or mutual information [9, 10]. Regression-based algorithms formulate network inference as a variable selection problem with the goal to search for candidate regulators (i.e., parent nodes) for each target gene, for example [11–13]. In particular, we previously showed the effectiveness of Bayesian Model Averaging (BMA) regression methods using time series data, in which snapshots of expression levels are taken at a few regular intervals after exposure to a drug perturbation [14]. Later work highlighted the ability of BMA to integrate external biological knowledge in the network building process to improve prediction accuracy [15]. Most recently, we have introduced the ScanBMA method for searching the model space, which significantly improves prediction accuracy and computational efficiency [16]. These BMA network inference methods are implemented in the networkBMA package [17] as part of Bioconductor [18].

Many implementations of network inference algorithms are only available as libraries or packages that require knowledge of a programming language. This limits the number of potential users in the biomedical community. For instance, users need to be familar with the R programming language to use the software implementations of network inference methods in Bioconductor such as *minet* [19], *Genenet* [20], *predictionet* [21], *TDARACNE* [22], *networkBMA* [17]. Relatively few options exist for researchers looking for easy-to-use network generation tools that do not require writing code. One notable example is GRN2SBML [23] which provides both a R package and a graphical user interface. GRN2SBML represents networks using the XML-based systems biology markup language (SBML) [24] and can be used in conjunction with three network inference algorithms including NetGenerator [25], TILAR [26] and ExTILAR [27].

As another example, Cyni Toolbox (http://www.proteomics.fr/Sysbio/CyniProject) is a Cytoscape app that offers several network inference algorithms based on correlation, mutual information, and other approaches. Cyni Toolbox leverages rich functionality offered by Cytoscape, a platform for visualizing complex networks [28]. Cytoscape allows users to load various types of interaction data sets for modeling and analysis and integrate them with additional metadata using a graphical interface. One of the most powerful features of Cytoscape is its extensibility. Dozens of apps provide extra functionality in areas such as network generation, data import, network analysis, and many more [29].

### Our contributions

In this paper, we present CyNetworkBMA, a Cytoscape app that brings the powerful features of networkBMA to a wider biomedical community. CyNetworkBMA offers an alternative, GUI-based way of running BMA network inference, without the need to write even a single line of code. To construct a network from expression data, the user simply needs to load input files into Cytoscape and select a few options from a dialog window. The application provides default values for parameters required by networkBMA, but users can override them using the advanced options dialog. The input data can represent static expression levels as well as time series. CyNetworkBMA can also provide an assessment of the generated network if reference regulator-target gene pairs are available from the literature or other data sources. CyNetworkBMA can generate a large number of common assessment statistics, such as sensitivity, specificity, precision and recall. It can also plot ROC and precision-recall curves for the inferred network model and export assessment results to a file.

**Fig. 1:**
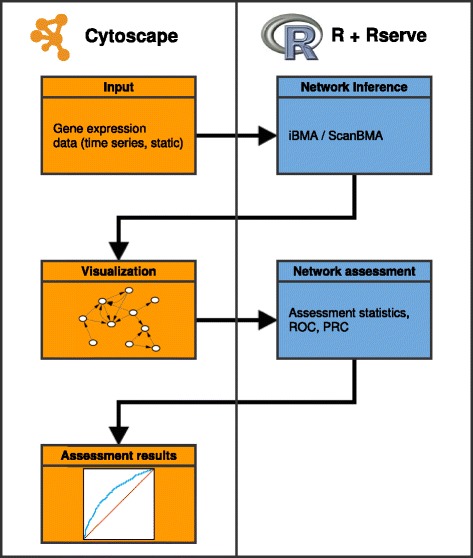
Network inference and assessment workflow. A diagram illustrating the full CyNetworkBMA application flow, from gene expression data to a generated network to assessment results

Figure 1 presents the overall application flow. A detailed user guide containing screen shots and step-by-step instructions for installing and using CyNetworkBMA is available as Additional file 1.

## Methods

CyNetworkBMA is implemented in Java as an OSGi bundle app compatible with Cytoscape 3.1.0 and later. It uses Rserve to integrate with R over a binary protocol on top of TCP/IP [30]. This means Cytoscape and R run in separate processes, potentially on different machines and platforms. CyNetworkBMA requires certain packages to be installed on the R instance: networkBMA for network inference and assessment, igraph [31] for algorithms used in removing potential cycles from networks, and Rserve for exposing R services over TCP/IP.

For large networks, the inference algorithm can run for a long time and it would be impractical to block Cytoscape until the execution finishes. Therefore, CyNetworkBMA runs each network inference job in a dedicated background thread. The user can use Cytoscape normally while a job is running. The app will display a notification when the job finishes or encounters an error. Multiple jobs can run in parallel at any given time. However, an R server running on Windows can handle only one connection at a time because of a limitation of Rserve implementation for that platform.

## Results and discussion

### Loading input data

We will demonstrate the functionality of CyNetworkBMA by using one of the data sets from DREAM4 In Silico Network Challenge [32–34], specifically, the time series file for the first network of size 100. This sample input file is available as Additional file 2. CyNetworkBMA requires input data sets to be in the form of unassigned Cytoscape tables, so the first step is loading the input file into a table. Each row in a Cytoscape table has to have a unique key. If an input data file does not have a key column, we must add it before the file can be imported. Keys can be of any data type as long as each value is unique. A simple sequence number is sufficient. CyNetworkBMA assumes that input data contain gene expression measurements only. If a file contains additional columns (e.g., time points), the user has the option to exclude these columns at the time of import or in the network inference step.

### Network inference

**Fig. 2:**
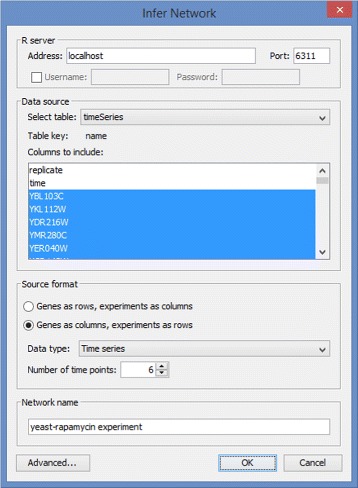
Main network inference dialog. The main inference dialog that lets the user specify connection parameters, source table, and the format of input data. A sample input data corresponding to this example is available as Additional file 2

**Fig. 3:**
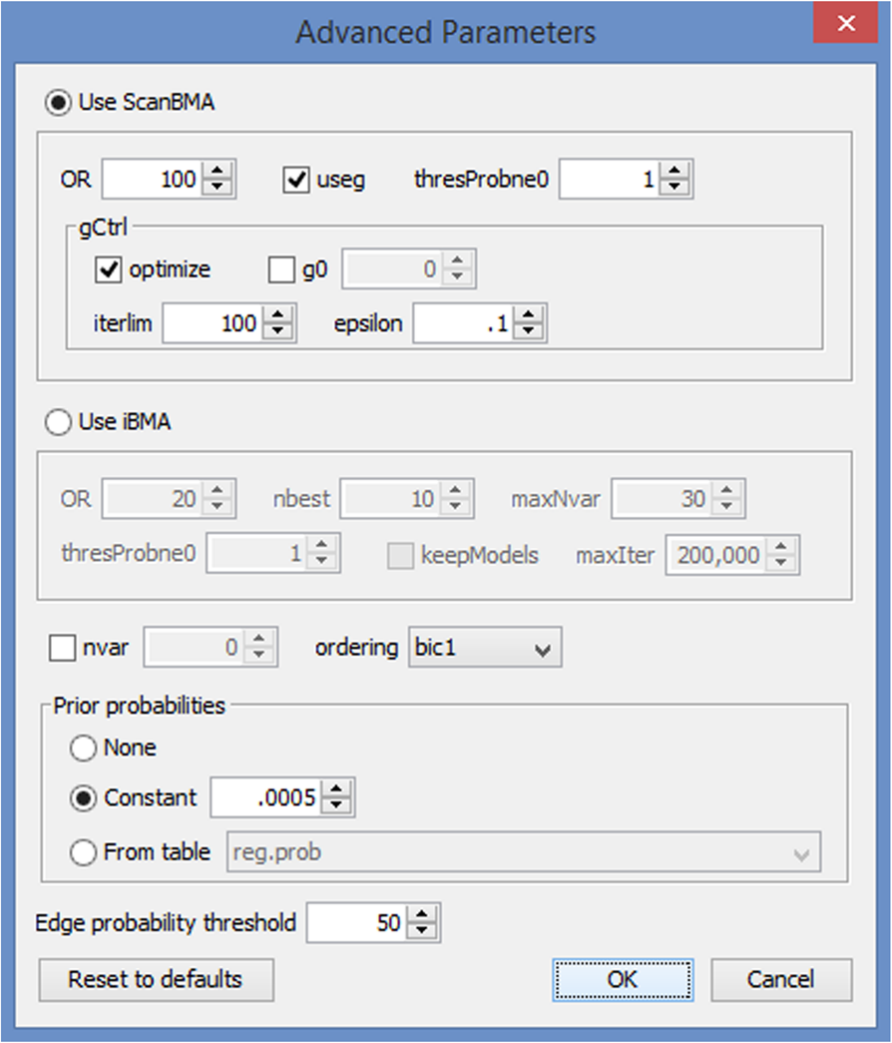
Advanced options dialog. The advanced parameters for fine-tuning the execution of the BMA algorithm

Once the file is loaded, we select the network inference option from the main menu. This produces a dialog window that allows us to choose the data source and specify the input format (see Fig. 2). At this time, the user also has the option to choose columns used in the analysis. In the DREAM4 example file, columns represent genes and rows are organized into 10 time series, each with 21 time points. CyNetworkBMA relies on the order of data points for implicit time information. We can view and change parameters controlling the BMA algorithm by going to the advanced dialog (see Fig. 3). The application provides default values that give reasonable compromise between the breadth of model search and execution time. However, in some cases, such as a particularly large data set, it may be beneficial to further restrict the search space.

The advanced dialog also allows the user to provide external information by specifying prior probabilities of regulatory relationships as a matrix. In the absence of prior probabilities of relationships between specific regulator-gene pairs, the user can specify a constant size prior, indicating the expected network density.

After we click OK on the main inference dialog, CyNetworkBMA submits a new job to the server whose address we specified. When the job is finished, the application will display the inferred network. In the meantime, we can use other features in Cytoscape normally. In the DREAM4 example, we run the inference algorithm with default parameters. The computation takes under 20 s on acomputer with dual-core Intel 2.5 GHz CPU and 4 GB of memory. The resulting network has 97 nodes and 172 directed edges. CyNetworkBMA calculates the in- and outdegree of each node and stores them in a node table. It also provides the posterior probability of each edge in the network. In our example, we thresholded the esdges at 50 %, so the posterior probabilities of the inferred edges are between 0.5 and 1.

### Network assessment

DREAM4 also provides the underlying true networks used to generate expression data (“gold standard”). CyNetworkBMA can leverage such reference information to assess the quality of a predicted network. To use this feature, we first generated a new network from the file we used before, this time setting the posterior probability threshold in the advanced options dialog to 0. The resulting network now contained many more edges, with a majority of them having very low probabilities. Note that the Occam’s window algorithm used returns posterior probabilities equal to zero for many edges, because as an approximation edges with very low posterior probabilities are excluded. Thus a posterior probability threshold of zero is effectively a very small positive threshold, determined by the control parameters of the Occam’s window algorithm.

We can import the gold standard as a text file into Cytoscape. The assessment feature in CyNetworkBMA accounts for incomplete knowledge in which the true underlying network is not fully known. This is almost always the case with real data, in which only a subset of interactions is documented in the literature. However, in the case of synthetic data such as DREAM4, the user should make sure that all nonexistent edges in the reference network are removed before import.

**Fig. 4:**
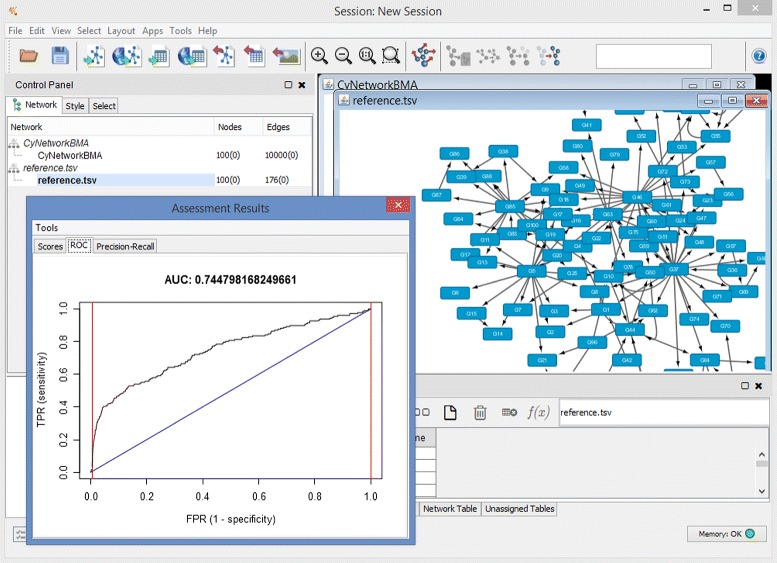
Network assessment tool. An example ROC curve generated by CyNetworkBMA with a network visualization in the background

Once the reference network is loaded, we open the assessment dialog and select the inferred network and the reference from their respective drop-down lists. Every edge in our network under assessment has a posterior probability assigned to it. When a network does not have such probabilities on the edges, the application assumes all edges have probabilities equal to 1. After we click OK, CyNetworkBMA runs the assessment function and presents a window with three tabs (Fig. 4). The first tab shows various assessment statistics for a given probability threshold, the value of which can be changed by moving a slider. The user can export the underlying data to a Cytoscape table, from where they can be saved to a file (see Additional file 3). The other two tabs show ROC and precision-recall curves, respectively, and their corresponding area under curve (AUC). The curves can also be exported to an image file. Our example network has an area under ROC curve of around 0.74. For networks inferred using the other four 100-gene data sets from DREAM4, this value ranges from 0.65–0.72. Table 1 shows other assessment scores for the example network.

**Table 1:** Selected assessment measures for a network generated from the example DREAM4 data set

Cutoff	50 %	95 %	99 %
Accuracy	0.9478	0.9504	0.9507
Precision	0.45	0.5085	0.52
Recall	0.2045	0.1705	0.1477
F1 score	0.2813	0.2553	0.2301

### Performance evaluation

We compared the performance of our BMA network inference methods to other leading methods in the literature [14–16]. In particular, we evaluated the performance of our network inference methods, ScanBMA and iBMA, using both yeast data and simulated data. On a time series yeast data, we showed that ScanBMA produced the highest accuracy and area under the ROC curve, while iBMA produced the highest area under the precision-recall curves when compared to another multivariate variable selection method (LASSO as implemented in the R package glmnet [35]), as well as several mutual information based methods (CLR, MRNET and ARACNE as implemented in the Bioconductor package minet [19]). On the simulated DREAM4 time series data consisting of 10 genes [32–34], we showed that ScanBMA again outperformed LASSO, CLR, MRNET, ARACNE in addition to Bayesian networks in terms of the area under the ROC and precision-recall curves. Please refer to Tables 1, 4, 5 in Young et al. for details.

Our latest software tool, CyNetworkBMA, implements the same network inference methods (ScanBMA, iBMA) at the back-end, while adding a graphical user interface to the front-end. CyNetworkBMA allows the user to leverage the functional capabilities of cytoscape, including visualizing large complex networks and integrating networks with annotations.

## Conclusions

We have developed CyNetworkBMA to make BMA network inference accessible to a wide user base by integrating networkBMA with Cytoscape. CyNetworkBMA takes advantage of Cytoscape’s support for multiple platforms, including Microsoft Windows, Linux and Mac OS X. The BMA algorithm itself can run on a local or remote R server. Multiple users can therefore submit jobs to a central server without having to install R on their machines. On the other hand, a single user can submit different jobs to different servers for parallel execution. Our application addresses both the usability and scalability of inferring gene networks from omics data.

## Availability and requirements

- **Project name:** CyNetworkBMA
- **Project home page:**http://webdatascience.github.io/CyNetworkBMA
- **Operating system(s):** Platform independent
- **Programming language:** Java, R
- **Other requirements:** Cytoscape 3.1.0 or higher, R 3.0 or higher, Java 1.6 or higher, networkBMA package from Bioconductor, Rserve and igraph packages from CRAN installed.
- **License:** GNU GPL v2
- **Any restrictions to use by non-academics:** None

We tested CyNetworkBMA on the following operation systems: Mac OS 10.8, 10.9, Windows 8, Windows 10, Ubuntu 12.04 and 14.04. Our latest testing includes Cytoscape 3.2.1, Java 1.8, R 3.2.1.

## Additional files

Additinal file 1
**User Manual.** This document provides screen shots and step-by-step guide for installing and using CyNetworkBMA. (PDF 157 kb)

Additinal file 2
**Sample input data file.** This is a text file containing the dataset “ *i* *n* *s* *i* *l* *i* *c* *o*_*s* *i* *z* *e*100_1_*t* *i* *m* *e* *s* *e* *r* *i* *e* *s*.*t* *x* *t*” was derived from DREAM 4 Challenge 2 [32, 33], and is publicly available from our project home page (http://webdatascience.github.io/CyNetworkBMA). (TXT 204 kb)

Additinal file 3
**Generated edge list.** The CyNetworkBMA output consisting of a list of edges and their probabilities that represent a gene network generated from the example DREAM4 input data set (available as Additional file 2). This output file corresponding to Additional file 3 is a text file consisting of three columns: parent, child, posterior probabilities. Each line represents a directed edge from parent node to child node with the posterior probability in the third column. We provide this file so that the user can verify the examples shown in this manuscript. (CSV 345 kb)
