# Supplementary material for: CyNetworkBMA: a Cytoscape app for inferring gene regulatory networks
Source: Source Code Biol Med. 2015 Nov 11;10:11. doi: 10.1186/s13029-015-0043-5 (PMC4642660; doi:10.1186/s13029-015-0043-5)
Supplement: Additinal file 1 — User Manual. This document provides screen shots and step-by-step guide for installing and using CyNetworkBMA. (PDF 157 kb) [file 13029_2015_43_MOESM1_ESM.pdf]

CyNetworkBMA: User Manual

Authors: Maciej Fronczuk, Adrian Raftery, Ka Yee Yeung

CyNetworkBMA is a Cytoscape app for inferring gene regulatory networks from expression data using the regression-based Bayesian Model Averaging (BMA) algorithm. Internally, it uses functionality provided by the networkBMA package.

Installation

Downloading and installing the app

CyNetworkBMA requires Cytoscape version 3.1.0 or later. You can download Cytoscape from www.cytoscape.org.

CyNetworkBMA is available on Cytoscape App Store and can be installed using the App Manager (Apps → App Manager).

Installing R

You will need to have R installed on the local machine or a remote server. R can be downloaded from www.r-project.org.

Installing R packages (Bioconductor, networkBMA, Rserve and igraph)

In R, run:

```
source("http://bioconductor.org/biocLite.R")
biocLite("networkBMA")

install.packages(c("Rserve","igraph"))
```

If you run R on a remote machine, be sure to enable remote access in Rserve and configure the firewall accordingly.

Starting the R server

Start R and type:

```
library(Rserve)
Rserve()
```

Inferring a Network

Load your input data into a table by going to File → Import → Table → File... (sample files can be found here). The file must have a key column and each row has to have a unique identifier (key). In the dialog window, choose the option to import data into an unassigned table.

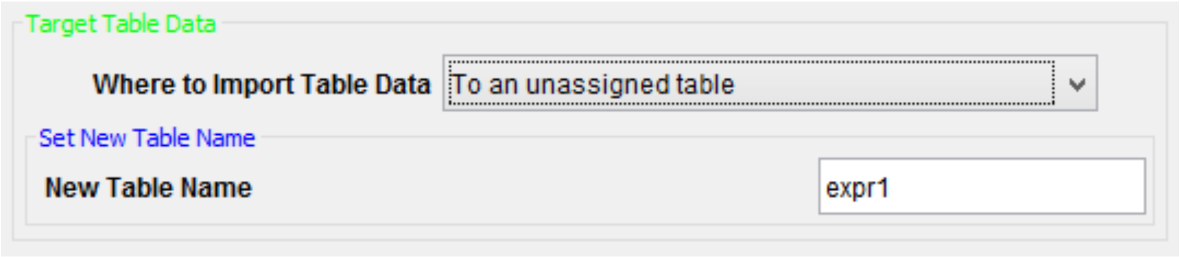

To run the network inference algorithm, go to Apps → CyNetworkBMA → Infer Network.... A dialog window will pop up.

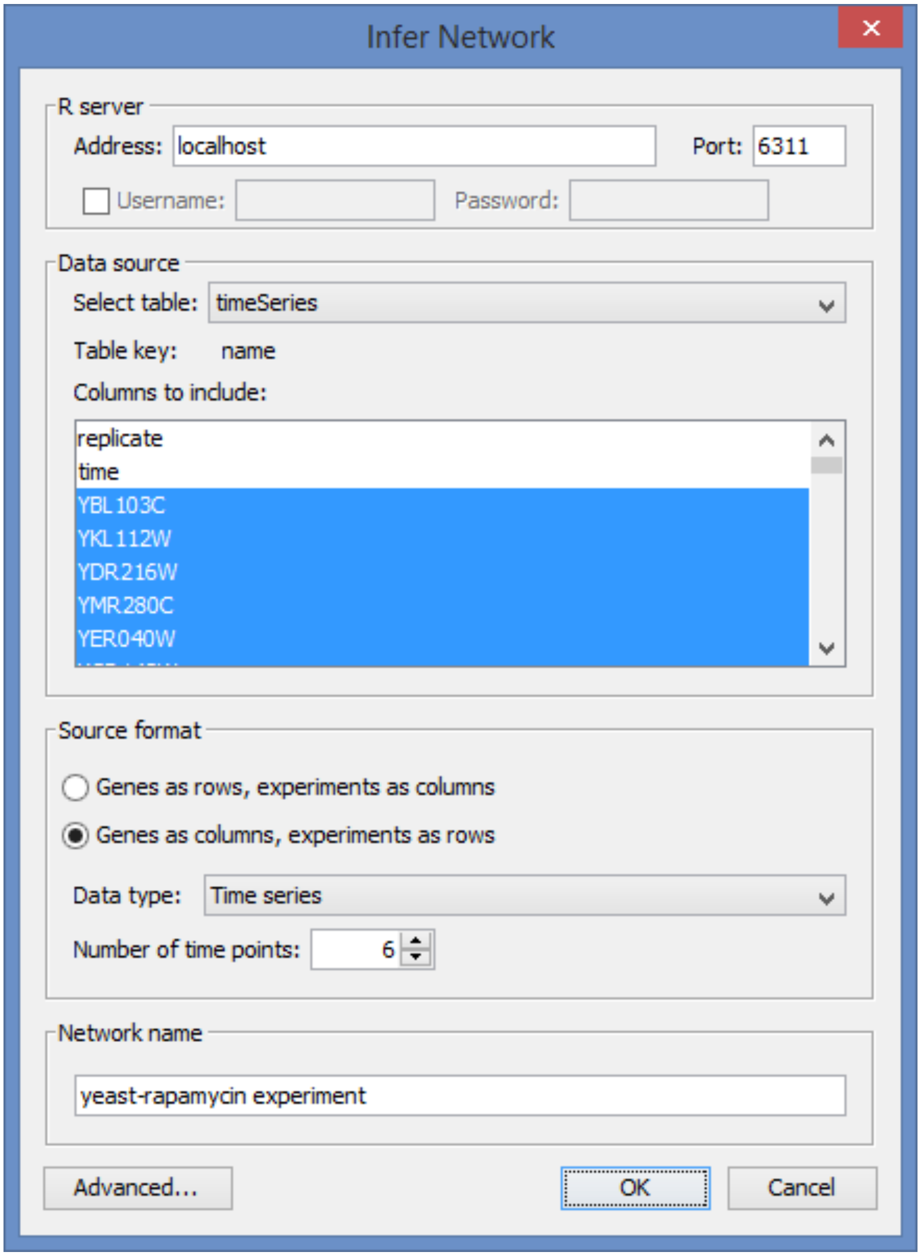

R server
Connection parameters for the R server. If you run a local R server, leave the default parameters unchanged. If your server requires authentication, select the checkbox and provide username and password.

Data source
Table and the columns to provide as an input to the algorithm. By default, all non-key columns in the table are used.

Source format
Format of the input table. If the table contains time series data, the number of rows should be a multiple of the number of time points, and rows should be grouped by a cell line or replicate. Each group should contain the same number of ordered time points.

Network name
The name of the network as will appear in Cytoscape.

Running the inference algorithm
When you click OK, CyNetworkBMA will create a job and submit it to the R server. You can use Cytoscape while the job is running. The app will notify you upon job completion. You can see all in-progress and finished jobs by going to Apps → CyNetworkBMA → Show Jobs.... It is possible to have more than one job running at the same time. However, an R server running on Windows can handle only one connection at a time because the Rserve implementation for that platform is more limited. At present, it is not possible to terminate a running job from Cytoscape, but you can kill the Rserve process that runs the algorithm on the R side (on Windows, closing R will have the same effect).

Advanced Options
This dialog lets you fine-tune parameters passed to networkBMA when defaults suggested by the app are not appropriate (e.g. when the input file is particularly large).

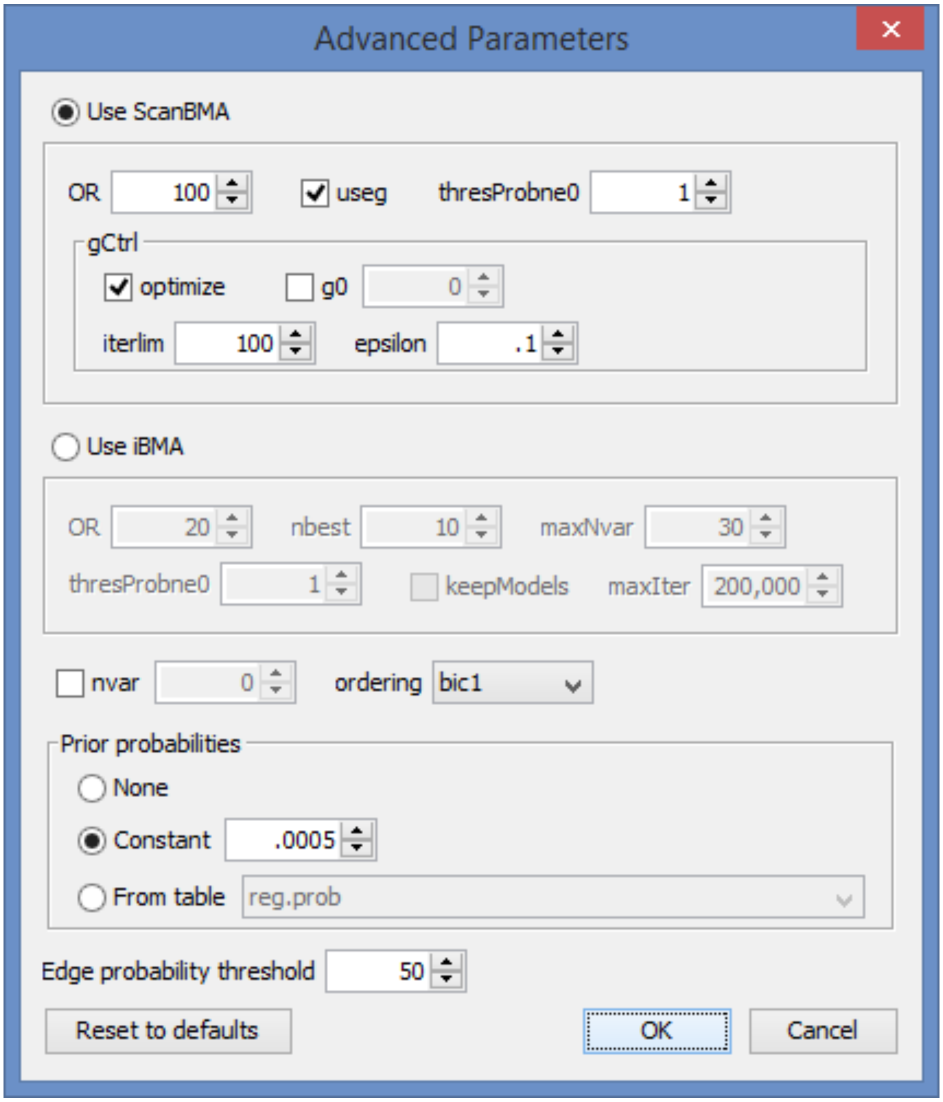

ScanBMA
ScanBMA is an efficient algorithm for searching model space that uses the Occam's window principle to quickly discard models with low predictive quality (Young et al., 2014). Input parameters for ScanBMA are as follows:

- *OR*: A number specifying the maximum ratio for excluding models in Occam's window.
- *useg*: Indicates whether to use Zellner's g-prior in model likelihood evaluation. If disabled, ScanBMA will use BIC to approximate the likelihood.
- *thresProbne0*: Threshold (in percent) for the posterior probability that each variable is has a non-zero coefficient (in percent). Variables with posterior probability less than thresProbne0 are removed in future BMA iterations.
- *optimize*: Indicates whether to optimize g using an iterative EM algorithm or use a fixed value of g.
- *g0*: An initial value of g to use if optimize is enabled, or the fixed value to use without optimization.
- *iterlim*: The maximum number of iterations of the EM algorithm to use.
- *epsilon*: The precision with which to find g using the EM algorithm.

iBMA
Iterative BMA is an older heuristic for selecting the best set of models (Lo et al., 2012). Input parameters for iBMA are as follows:

- *OR*: A number specifying the maximum ratio for excluding models in Occam's window.
- *nbest*: A positive integer specifying the number of models of each size to be considered by leaps-and-bounds in determining the model space for Bayesian Model Averaging.
- *maxNvar*: A positive integer specifying the maximum number of variables (excluding the intercept) used in each iteration of BMA.
- *thresProbne0*: Threshold (in percent) for the posterior probability that each variable is has a non-zero coefficient (in percent). Variables with posterior probability less than thresProbne0 are removed in future BMA iterations.
- *keepModels*: A logical value indicating whether or not to keep the BMA models from all of the iterations and apply Occam's window using OR at the end, or to apply Occam's window in all BMA iterations and return the final model. Enabling this option requires more memory and may slow the computation as a result.
- *maxIter*: A positive integer giving a limit on the number of iterations.

nvar and ordering
- *nvar*: The number of top-ranked (see ordering) genes to be considered in the modeling. If the checkbox is not selected, all genes will be considered. Setting a large value will increase computation time.
- *ordering*: The ordering to be used for the genes or variables.

Prior probabilities
- *None*: No prior information will be used in modeling the network.
- *Constant:* The probability of a regulator-gene pair in the network.
- *From table*: A matrix in which the (i,j) entry is the estimated prior probability that gene i regulates gene j.

Edge probability threshold
The minimum posterior probability (in percent) for an edge to be included in the final network.

Assessing a Network
CyNetworkBMA can generate various assessment statistics for the inferred network if a reference network is available from another source (e.g. curated database or literature). To run network assessment, load the reference network (File → Import → Network → File...) and go to Apps → CyNetworkBMA → Assess Network....

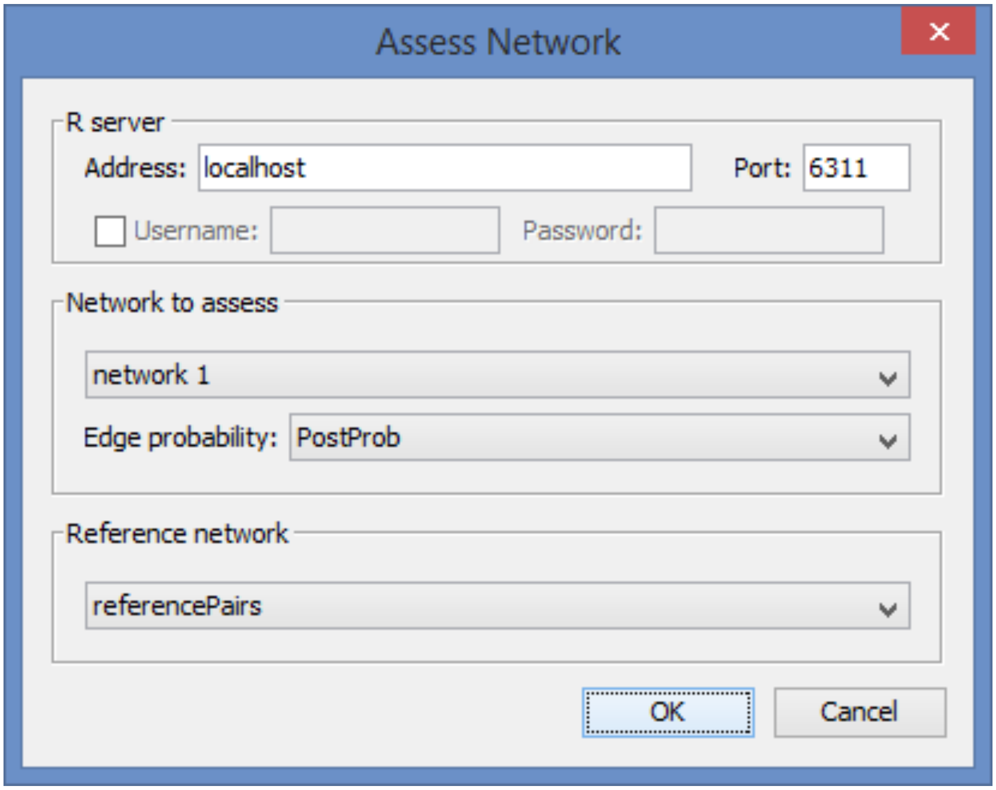

R server
Connection parameters for the R server.

Network to assess
The inferred network to assess and the name of the column in the edge table that corresponds to edge probability. If edges in the network have no probability attribute, the probability is assumed to be 1.

Reference network
The network consisting of known regulatory relationships.
When you click OK, CyNetworkBMA will run the assessment algorithm and display a results window once finished.

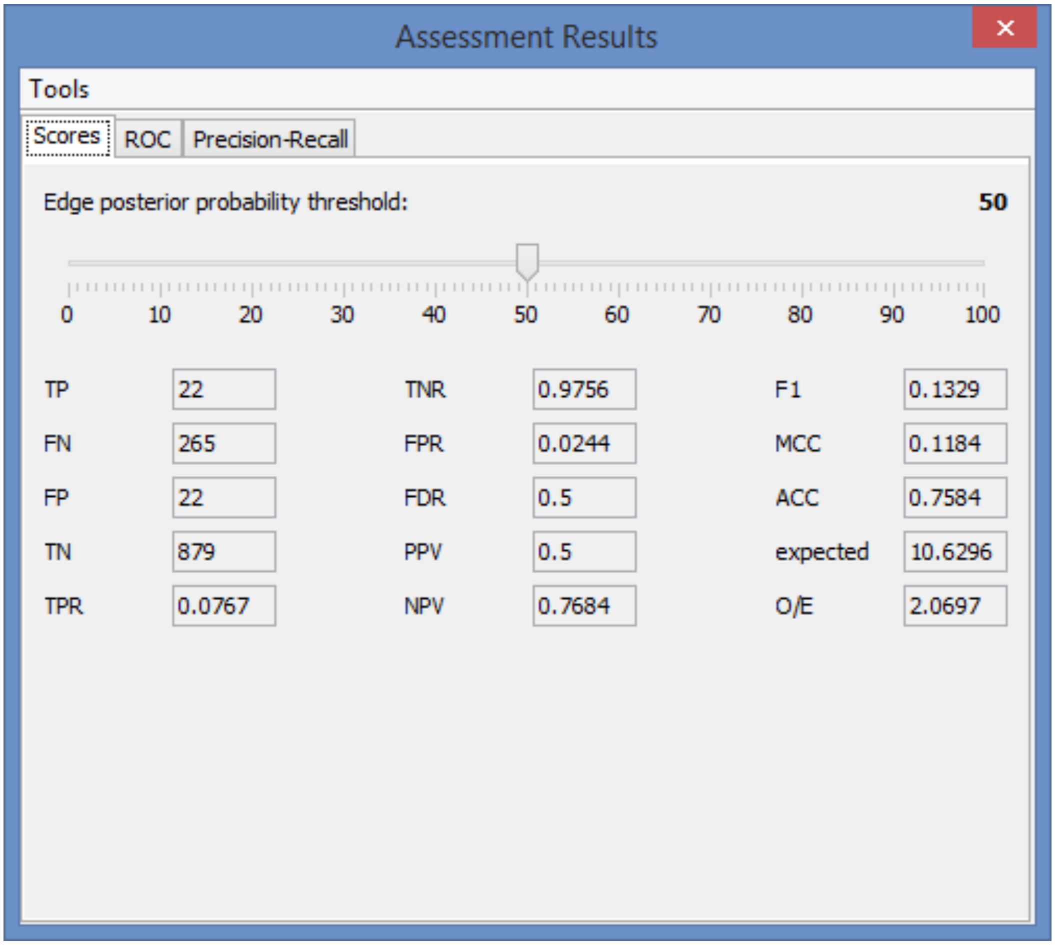

The slider lets you vary the probability threshold above which an edge is included in the network under assessment. You can export the raw scores corresponding to each distinct edge probability in the network by going to Tools → Save Current Tab....

The other two tabs show the ROC (receiver operating characteristic) and precision-recall curves. You can export each curve to an image file by using the Save Current Tab... function.
